# Cell aggregation promotes pyoverdine-dependent iron uptake and virulence in *Pseudomonas aeruginosa*

**DOI:** 10.3389/fmicb.2015.00902

**Published:** 2015-08-28

**Authors:** Daniela Visaggio, Martina Pasqua, Carlo Bonchi, Volkhard Kaever, Paolo Visca, Francesco Imperi

**Affiliations:** ^1^Department of Biology and Biotechnology “Charles Darwin”, Sapienza University of RomeRome, Italy; ^2^Department of Sciences, Universita degli Studi Roma TreRome, Italy; ^3^Research Core Unit Metabolomics, Institute of Pharmacology, Hannover Medical SchoolHannover, Germany; ^4^Pasteur Institute – Cenci Bolognetti Foundation, Sapienza University of RomeRome, Italy

**Keywords:** cell aggregates, extracellular polysaccharide, gene regulation, iron uptake, mechanosensor, *Pseudomonas aeruginosa*, siderophore, virulence

## Abstract

In *Pseudomonas aeruginosa* the Gac signaling system and the second messenger cyclic diguanylate (c-di-GMP) participate in the control of the switch between planktonic and biofilm lifestyles, by regulating the production of the two exopolysaccharides Pel and Psl. The Gac and c-di-GMP regulatory networks also coordinately promote the production of the pyoverdine siderophore, and the extracellular polysaccharides Pel and Psl have recently been found to mediate c-di-GMP-dependent regulation of pyoverdine genes. Here we demonstrate that Pel and Psl are also essential for Gac–mediated activation of pyoverdine production. A *pel psl* double mutant produces very low levels of pyoverdine and shows a marked reduction in the expression of the pyoverdine-dependent virulence factors exotoxin A and PrpL protease. While the exopolysaccharide-proficient parent strain forms multicellular planktonic aggregates in liquid cultures, the Pel and Psl-deficient mutant mainly grows as dispersed cells. Notably, artificially induced cell aggregation is able to restore pyoverdine-dependent gene expression in the *pel psl* mutant, in a way that appears to be independent of iron diffusion or siderophore signaling, as well as of recently described contact-dependent mechanosensitive systems. This study demonstrates that cell aggregation represents an important cue triggering the expression of pyoverdine-related genes in *P. aeruginosa*, suggesting a novel link between virulence gene expression, cell–cell interaction and the multicellular community lifestyle.

## Introduction

*Pseudomonas aeruginosa* is a metabolically versatile Gram-negative bacterium and an opportunistic pathogen in cystic fibrosis (CF) and otherwise critical patients, causing both chronic and acute infections (Driscoll et al., 2007). The ability of *P. aeruginosa* to rapidly adapt to diverse ecological niches and to switch from acute to chronic infections is related to the tightly regulated expression of specific sub-sets of genes in response to environmental cues (Stover et al., 2000). Characteristic traits of *P. aeruginosa* chronic infection are the microcolony and biofilm mode of growth (Parsek and Singh, 2003). Microcolonies are small aggregates of cells that open the way to the communal organization of biofilms, which are surface-associated communities of bacteria encased in a self-generated polymeric matrix (Zhao et al., 2013). Extracellular polysaccharides represent a key component of the *P. aeruginosa* biofilm matrix, and are involved in surface attachment and cell–cell interaction (Ma et al., 2009).

*Pseudomonas aeruginosa* strains can produce three main exopolysaccharides, namely alginate, Pel, and Psl (Colvin et al., 2012). Alginate confers the typical mucoid phenotype to producing strains (Sherbrock-Cox et al., 1984), and plays a crucial role in CF lung colonization. Psl and Pel are normally produced by non-mucoid strains, and failure to produce Pel and/or Psl exopolysaccharides impairs biofilm formation *in vitro* (Colvin et al., 2012). The exopolysaccharide Psl consists of repeating pentamers of D-mannose, L-rhamnose, and D-glucose (Ma et al., 2007). The helical distribution of Psl on the cell surface promotes cell–cell and cell–surface interactions in microcolonies (Zhao et al., 2013). Psl also plays an essential role in the maintenance of the mature biofilm structure (Jackson et al., 2004; Ma et al., 2009). The exopolysaccharide Pel was originally identified by a transposon mutagenesis screening for the loss of surface pellicle formation in *P. aeruginosa* PA14 (Friedman and Kolter, 2004). Pel structure has not yet been determined, although carbohydrate analysis suggested a glucose-rich composition (Friedman and Kolter, 2004).

The production of the Pel and Psl exopolysaccharides is controlled by many regulatory networks at the level of transcription, translation, and biosynthesis (Goodman et al., 2004; Ventre et al., 2006; Lee et al., 2007; Sakuragi and Kolter, 2007; Gilbert et al., 2009). The Gac system and the signaling molecule cyclic diguanylate (c-di-GMP) are the best characterized regulatory networks involved in the regulation of *pel* and *psl* gene expression and, consequently, in the switch from the planktonic to the biofilm lifestyle. The Gac system relies on the sensor kinase GacS that, in response to a still unknown signal, activates the transcriptional regulator GacA, which in turn promotes the transcription of two small non-coding RNAs (sRNAs), RsmZ and RsmY (Brencic et al., 2009). These sRNAs bind to and sequester the mRNA binding protein RsmA, thereby inhibiting its activity as translational repressor (Heeb et al., 2006). While *psl* gene expression is directly repressed by RsmA at the translational level (Irie et al., 2010), there is no evidence of a direct effect of RsmA on the *pel* genes, although transcriptomic analysis showed that the active state of the Gac network (i.e., *rsmA* mutation) also promotes transcription of the *pel* operon (Brencic and Lory, 2009). The Gac system has recently been shown to positively affect the intracellular levels of the signaling molecule c-di-GMP, which also induces exopolysaccharides production (Moscoso et al., 2011, 2014; Irie et al., 2012; Frangipani et al., 2014). This second messenger regulates Pel production both at the transcriptional and post-transcriptional level, by inhibiting the activity of the transcriptional repressor FleQ (Hickman and Harwood, 2008), and activating the Pel biosynthesis protein PelD (Lee et al., 2007). Although the molecular mechanism has not been elucidated yet, high intracellular levels of c-di-GMP also increase the expression of *psl* genes (Hickman et al., 2005).

Besides exopolysaccharides production and biofilm formation, Gac and c-di-GMP also act in a concerted way to promote the expression of pyoverdine genes (Frangipani et al., 2014), and it has recently been reported that the exopolysaccharides Pel and Psl are important for c-di-GMP regulation of pyoverdine production (Chen et al., 2015). Pyoverdine is a green fluorescent siderophore which plays a prominent role in *P. aeruginosa* pathogenicity (Visca et al., 2007; Cornelis and Dingemans, 2013). Pyoverdine acts not only as a high-affinity iron scavenger, but also serves as a signal molecule to promote the expression of important *P. aeruginosa* virulence factors, *via* a cell–surface signaling cascade which involves the other membrane ferri-pyoverdine receptor FpvA and the cytoplasmic membrane-spanning antisigma factor FpvR, ultimately leading to the activation of the extracytoplasmic function (ECF) sigma factor PvdS (Lamont et al., 2002; Llamas et al., 2014). PvdS directs the transcription of almost thirty *P. aeruginosa* genes, including those involved in pyoverdine biosynthesis and transport, the gene for the extracellular protease PrpL and, indirectly, the exotoxin A gene *toxA* (Ochsner et al., 2002; Llamas et al., 2014). The dual function of pyoverdine in iron uptake and virulence renders this siderophore essential for *P. aeruginosa* infectivity, as demonstrated in different mouse models (Meyer et al., 1996; Takase et al., 2000; Imperi et al., 2013). As for any iron-uptake system, pyoverdine production needs to be promptly shut down when intracellular iron levels are sufficiently high. This iron-mediated control occurs through the ferric uptake regulator Fur, an iron-sensing transcriptional repressor which binds to its co-repressor Fe^2+^ and inhibits transcription of the sigma factor gene *pvdS* (Ochsner et al., 2002). Besides Fur-Fe^2+^ mediated repression, pyoverdine production was found to be influenced by a number of environmental signals and regulatory pathways, including oxygen and nutrient availability, cellular communication and oxidative stress (reviewed in Llamas et al., 2014).

In the present study we demonstrate that the extracellular polysaccharides Pel and Psl are also essential for Gac-mediated regulation of pyoverdine production, which appears strongly repressed in a *pel psl* double mutant. We also provide evidence that artificially induced cell aggregation is able to restore pyoverdine-dependent gene expression in the Pel and Psl-deficient mutant. Our findings support the hypothesis that cell aggregation, rather than polysaccharide production *per se*, is an important cue triggering production of pyoverdine and pyoverdine-controlled virulence factors in *P. aeruginosa*.

## Materials and Methods

### Bacterial Strains, Growth Conditions, and Plasmids

Bacterial strains and plasmids used in this study are listed in Supplementary Table S1. The iron-depleted complex medium TSBD (Ohman et al., 1980) or the M9 minimal medium supplemented with 20 mM sodium succinate (Sambrook et al., 1989) were used as iron-poor media, to which FeCl_3_ was added at the indicated concentrations when required. Virulence factor production and gene expression assays were performed on bacteria grown at 37°C in 96-well microtiter plates (250 μl of medium in each well) under static conditions, unless otherwise stated. When specified, TSBD medium was supplemented with L-arabinose, agar, phytagel, or sucrose (Sigma–Aldrich) at the indicated final concentrations. Polystyrene beads (Polybead^®^ Microspheres 3.00 μm, Polysciences) were washed several times with sterile water and then resuspended in TSBD at the desired concentrations.

### Generation of Deletion and Conditional Mutants

All the primers and restriction sites used for PCR and cloning are listed in Supplementary Table S2. Deletion mutants in *pelABCD*, *pslABCD*, *fpvR*, *pilY1*, or *pilA* were generated using specific derivatives of the pDM4 suicide plasmid (Supplementary Table S1). The *fur* conditional mutant was generated using a recently described strategy (Lo Sciuto et al., 2014), based on the mini-CTX1-mediated insertion of the *fur* coding sequence under the control of an arabinose-dependent promoter into a neutral site of the *P. aeruginosa* genome, followed by the in-frame deletion of the endogenous *fur* gene using the pDM4Δ*fur* suicide plasmid (Supplementary Table S1), under permissive conditions (i.e., arabinose-containing media). Gene replacements and insertions were verified by PCR and DNA sequencing.

### Growth and Pyoverdine Measurements

Growth was measured as the OD_600_ of appropriate dilutions of bacterial cultures in sterile growth medium. Pyoverdine production was measured as the OD_405_ of culture supernatants appropriately diluted in 0.1 M Tris-HCl (pH 8), and normalized to the OD_600_ of the corresponding cultures (Imperi et al., 2009). When indicated, pyoverdine production was normalized to the number of colony forming units/ml.

### β-Galactosidase and c-di-GMP Assays

The β-galactosidase activity from *P. aeruginosa* cells carrying reporter plasmids (Supplementary Table S1) was determined spectrophotometrically using *o*-nitrophenyl-β-D-galactopyranoside as the substrate, normalized to the OD_600_ of the bacterial culture and expressed in Miller (1972) units. Intracellular c-di-GMP levels were determined by liquid chromatography coupled with tandem mass spectrometry as described (Spangler et al., 2010), and normalized to the corresponding cellular protein content determined using the DC protein assay kit (Bio-Rad) and bovine serum albumin as the standard.

### Western Blot Analysis of ToxA and PrpL Enzymatic Assay

For detection of ToxA, 100-μl aliquots of culture supernatants were supplemented with 20 μl of 6× SDS-PAGE loading dye [375 mM Tris-HCl (pH 6.8), 9% SDS, 50% Glycerol, 0.03% Bromophenol blue]. To normalize the amount of secreted proteins to bacterial growth, the volume of supernatants loaded into SDS-PAGE gels was calculated according to the formula: loading volume (μl) = 10/OD_600_ of the corresponding bacterial culture. Proteins resolved by SDS-PAGE were electrotransferred onto a nitrocellulose filter (Hybond-C extra, Amersham), and probed for ToxA using a rabbit polyclonal anti-ToxA antibody (Sigma–Aldrich). Filters were developed with 5-bromo-4-chloro-3-indoyl-phosphate and nitro blue tetrazolium chloride reagents for colorimetric alkaline phosphatase detection (Promega).

PrpL activity was determined as previously described (Imperi et al., 2010), using the chromogenic substrate Chromozym PL (tosyl-Gly-Pro-Lys-*p*-nitroanilide; Sigma–Aldrich) which is specific for PrpL (protease IV) and is not cleaved by other *P. aeruginosa* proteases (Caballero et al., 2001). Briefly, 10 μl of culture supernatants were mixed with 10 μl of 2 mg/ml Chromozym PL and 180 μl of phosphate buffer (pH 7.0) in 96-well microtiter plates. The OD_410_ was read at 2 min intervals for 30 min in a Victor^3^V plate reader (Perkin-Elmer), and the change in optical density (ΔOD_410_) per minute was determined. PrpL activity units were determined as: (ΔOD_410_ × total assay volume/ml)/(sample volume × E410 × light path); where total assay volume was 200 μl, sample volume was 10 μl, the extinction coefficient (E) of the product (*p*-nitroaniline) at 410 nm was 9.75, and the light path was 0.35 cm. PrpL activity was then normalized to the OD_600_ of the corresponding bacterial culture.

### Quantitative Real-Time Reverse-Transcription PCR (qRT-PCR)

Total RNA was purified by using RNeasy minicolumns, treated with DNase and re-purified with the RNeasy MinElute cleanup kit (Qiagen). cDNA was reverse transcribed from 0.5 μg of total RNA with PrimeScript RT reagent Kit (TaKaRa). cDNA was then used as the template for qRT-PCR in a 7300 Real-Time PCR System (Applied Biosystems) using SYBR green with the ROX detection system (Bio-Rad). The primers used for qRT-PCR are listed in Supplementary Table S2. At least three wells were run for each cDNA sample. Relative gene expression with respect to the housekeeping gene *rpsL* was calculated using the 2^-ΔΔCt^ method (Livak and Schmittgen, 2001).

### Confocal Microscopy

Thirty microliter of *P. aeruginosa* cultures in TSBD or M9 were spotted on a glass slide that was freshly coated with 0.5% agarose in water, and covered with a cover slip. Images were acquired with a Leica TCS SP5 inverted confocal microscope equipped with a HCX PLAPO lamba blue 40X/1.25 OIL objective (Zeiss). Images were recorded using specific sets for GFP (excitation at 488 nm, emission window from 500 to 600 nm).

### Laser Diffraction Analysis (LDA)

Laser diffraction analysis was performed in a Mastersizer 2000 (Malvern Instrument, UK) as previously described (Schleheck et al., 2009) with minor modifications. In particular, the following settings were used: stirring speed 1000 rpm, laser intensity 79%, 5,000 size scan, Frauenhofer count mode. The amount of bacterial suspension was adjusted to result in an LDA-obscurity of 2–6%. Size distribution scans are quantified by LDA and expressed as volume percentage, corresponding to the volumetric contribution of each particle size class to the total volume of all particle size classes (Schleheck et al., 2009).

### Statistical Analysis

Statistical analysis was performed with the software GraphPad Instat, using One-Way Analysis of Variance (ANOVA), followed by Tukey–Kramer multiple comparison tests.

## Results

### Exopolysaccharide Production is Essential for Gac-Mediated Regulation of Pyoverdine Production

It has recently been demonstrated that the presence of the Pel and/or Psl polysaccharides is required for the ability of the second messenger c-di-GMP to positively regulate the production of the *P. aeruginosa* siderophore pyoverdine (Chen et al., 2015).

**FIGURE 1:**
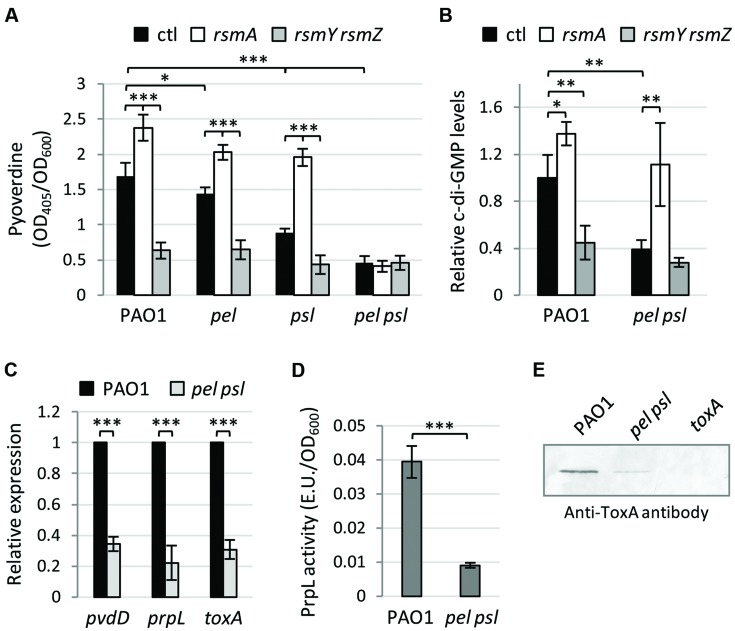
**The exopolysaccharides Pel and Psl are essential for Gac-mediated control on pyoverdine production and positively regulate pyoverdine-dependent virulence factors. (A)** Pyoverdine production by *Pseudomonas aeruginosa* PAO1, *pel*, *psl*, and *pel psl* mutants, and their derivatives deleted in the *rsmA* or in the *rsmY* and *rsmZ* genes. **(B)** Intracellular levels of c-di-GMP (relative to wild type PAO1) in *P. aeruginosa* PAO1, the *pel psl* mutant, and their derivatives deleted in the *rsmA* or the *rsmY* and *rsmZ* genes. **(C)** Relative mRNA levels of *pvdD*, *toxA*, and *prpL* as determined by qRT-PCR, and **(D)** PrpL enzymatic activity in culture supernatants of *P. aeruginosa* PAO1 and the *pel psl* mutant. **(E)** Western-blot showing ToxA levels in culture supernatants of *P. aeruginosa* PAO1, the *pel psl* mutant, and the *toxA* mutant (used as negative control). Bacteria were grown in TSBD at 37°C under static conditions for 14 h. Values in **(A–D)** are the mean (±SD) of at least three independent assays. Asterisks indicate statistically significant differences with respect to the corresponding parental strain (^∗^*p* < 0.05, ^∗∗^*p* < 0.01, ^∗∗∗^*p* < 0.001). The image in panel **(E)** is representative of four independent experiments giving similar results.

In order to verify whether the Pel and Psl exopolysaccharides are also involved in the Gac-mediated control of pyoverdine gene expression, we generated single and double deletion mutants in *pel* and *psl* genes in *P. aeruginosa* PAO1 (wild-type) and in isogenic mutants in which the Gac signaling is inactive (*rsmY rsmZ* mutant) or constitutively active (*rsmA* mutant; Supplementary Table S1). In agreement with previous observations (Frangipani et al., 2014), pyoverdine production in the iron-poor TSBD medium was higher in the *rsmA* mutant and strongly reduced in the *rsmY rsmZ* mutant compared with the wild type (WT; **Figure 1A**). This pyoverdine production profile was maintained in strains lacking either Pel or Psl, while pyoverdine production was drastically reduced in the *pel psl* double mutant irrespective of the activation state of the Gac system (**Figure 1A**). This response appeared to be independent of the previously reported effect of the Gac system on c-di-GMP production (Moscoso et al., 2011; Frangipani et al., 2014), since mass spectrometry analysis revealed that the Gac system is still able to control intracellular c-di-GMP levels in a Pel/Psl deficient background (**Figure 1B**). Thus, both the Gac system (**Figure 1A**) and the c-di-GMP second messenger (Chen et al., 2015) require at least one of two exopolysaccharides Pel and Psl to exert their control on pyoverdine biosynthesis, indicating that these exopolysaccharides, or an exopolysaccharide-dependent phenotype, are implicated in pyoverdine gene regulation. Indeed, we observed that the expression of the pyoverdine biosynthetic gene *pvdD* was greatly reduced in the *pel psl* mutant relative to the WT, as well as that of the pyoverdine-dependent virulence genes *toxA* and *prpL* (**Figure 1C**). Accordingly, the levels of the corresponding virulence factors in culture supernatants were significantly lower in the *pel psl* double mutant as compared to the WT strain (**Figures 1D,E**), as also observed for pyoverdine (**Figure 1A**). Therefore, lack of the Pel and Psl exopolysaccharides in *P. aeruginosa* causes a significant reduction not only in the production of the siderophore pyoverdine but also of pyoverdine-dependent virulence factors.

### Exopolysaccharides Affect Pyoverdine Production Independently of Fur and Pyoverdine Signaling

The presence/absence of extracellular polysaccharides on the bacterial cell surface could influence the transport of nutrients and/or signal molecules. Since pyoverdine gene regulation is directly or indirectly controlled by the intracellular levels of iron, through the iron sensor Fur, and by the availability of iron-loaded pyoverdine on the cell surface, through the pyoverdine signaling cascade, we investigated the role of Fur and pyoverdine signaling in the Pel and Psl control of pyoverdine-dependent gene expression.

**FIGURE 2:**
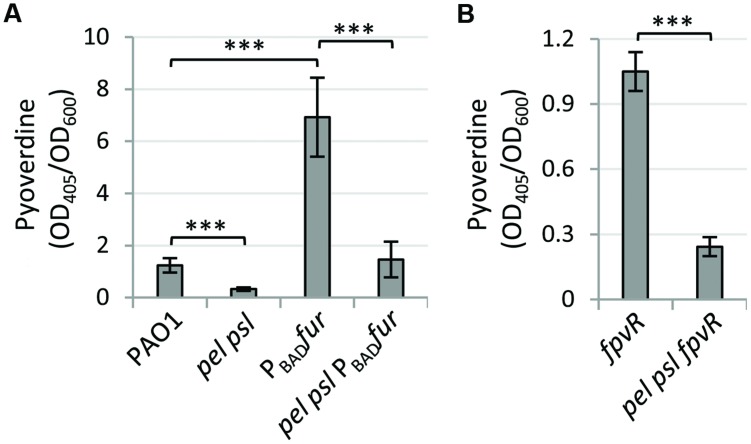
**Fur and pyoverdine signaling are not involved in exopolysaccharide-mediated pyoverdine regulation**. Pyoverdine production in **(A)** *P. aeruginosa* PAO1, the *pel psl* mutant, and their derivatives in which the native *fur* gene was replaced by an arabinose-inducible allele (P_BAD_*fur*), and **(B)** *P. aeruginosa* PAO1 *fpvR* and *pel psl fpvR* mutants. Bacteria were cultured in TSBD without arabinose at 37°C under static conditions for 14 h, and values are the mean (±SD) of at least three independent assays. Asterisks indicate statistically significant differences with respect to the corresponding parental strain (^∗∗∗^*p* < 0.001).

Since Fur is considered an essential protein in *P. aeruginosa* due to the inability to obtain *fur* null mutants in this species (Barton et al., 1996; Cornelis et al., 2009), to verify the involvement of Fur in exopolysaccharide-mediated control of pyoverdine production we generated a *fur* conditional mutants in WT and *pel psl* backgrounds, by replacing the native *fur* gene with an arabinose-inducible allele. The PAO1 *fur* conditional mutant grew poorly in the iron-poor TSBD medium, unless arabinose was added to the medium (Supplementary Figure S1). Compared with the WT strain, pyoverdine levels were significantly higher in the *fur* conditional mutant grown without arabinose, and were not shut down by the addition of iron to the growth medium (Supplementary Figure S1). This evidence confirmed the suitability of our conditional mutagenesis strategy to obtain Fur-depleted *P. aeruginosa* cells. Notably, the differences in pyoverdine production between *fur* and *pel psl fur* conditional mutants in the absence of arabinose were comparable to those observed for the WT and the *pel psl* mutant (**Figure 2A**), clearly indicating that Pel and Psl control pyoverdine gene expression in a way that is independent of Fur. Although not strictly instrumental in the present study, the *fur* conditional mutant generated in this work will represent a valuable tool for better understanding iron uptake regulation and metabolism in *P. aeruginosa*.

To test the effect of exopolysaccharides on pyoverdine signaling, we deleted the *fpvR* gene in both the WT and Pel- and Psl-negative backgrounds, in order to obtain strains in which PvdS activity cannot be influenced by pyoverdine signaling through the anti-sigma factor FpvR (Lamont et al., 2002; Tiburzi et al., 2008). Again, deletion of *fpvR* did not restore pyoverdine production in the *pel psl* mutant (**Figure 2B**), ruling out the involvement of FpvR and, thus, of the pyoverdine signaling cascade on the exopolysaccharides-mediated control of pyoverdine production.

### Artificially Induced Cell Aggregation Restores Production of Virulence Factors in Exopolysaccharide-Defective Cells

**FIGURE 3:**
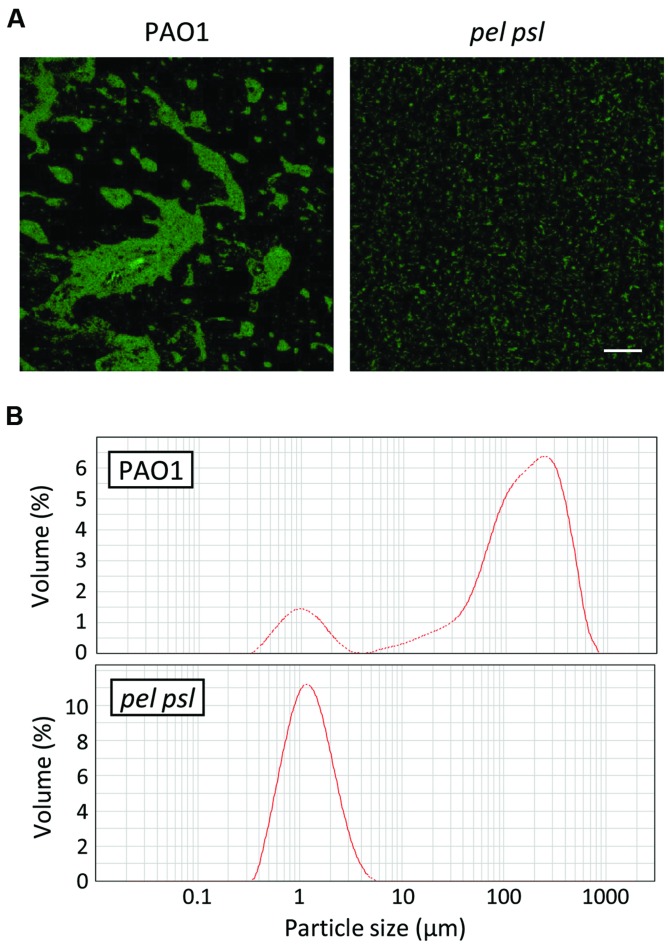
**Role of Pel and Psl exopolysaccharides in planktonic aggregation of *P. aeruginosa* cells. (A)** Confocal microscopy images of *P. aeruginosa* PAO1 and *pel psl* cells harboring the GFP-expressing vector pMMG cultured in TSBD at 37°C under static conditions for 14 h. The images are representative of several micrographs from five independent experiments. Bar: 50 μm. **(B)** Representative laser diffraction analysis (LDA) particle-size scans of *P. aeruginosa* PAO1 and *pel psl* liquid cultures in TSBD after 14 h of growth at 37°C.

Our findings argue for a prominent role of extracellular polysaccharides in triggering the expression of pyoverdine-dependent virulence genes. As shown in **Figure 1A**, each single exopolysaccharide has the ability to promote pyoverdine production, suggesting that a phenotype that depends on the presence of exopolysaccharide(s), rather than a specific exopolysaccharide molecule, could be important for pyoverdine-related gene expression. It is well known that extracellular polysaccharides have a role in surface attachment and biofilm formation (Colvin et al., 2012). However, exopolysaccharides have also been proposed to mediate planktonic aggregation (Klebensberger et al., 2007; Alhede et al., 2011), as confirmed by our observation that Pel and Psl-deficient cells prevalently grow in TSBD medium as dispersed cells, while Pel and Psl-proficient WT cells grow as large aggregates including hundreds of cells (**Figure 3A**). These bacterial aggregates appear quite loose, and can be partially dispersed by vigorous pipetting (data not shown). This qualitative evidence was confirmed by determining the size and distribution of planktonic cells and cell aggregates by LDA. This analysis revealed that cell aggregates with a size ranging from 30 to 600 μm represent more than 85% of the bacterial population in planktonic cultures of the WT strain. In contrast, cell aggregates were not detectable in planktonic cultures of the *pel psl* mutant, which only contained single cells (**Figure 3B**). Such planktonic aggregates were also observed in cultures of the WT PAO1 grown in M9 minimal medium, while they were not detected in *pel psl* cultures (Supplementary Figure S2). Notably, also in this medium pyoverdine production was strongly reduced in the *pel psl* mutant with respect to the WT strain (Supplementary Figure S2).

**FIGURE 4:**
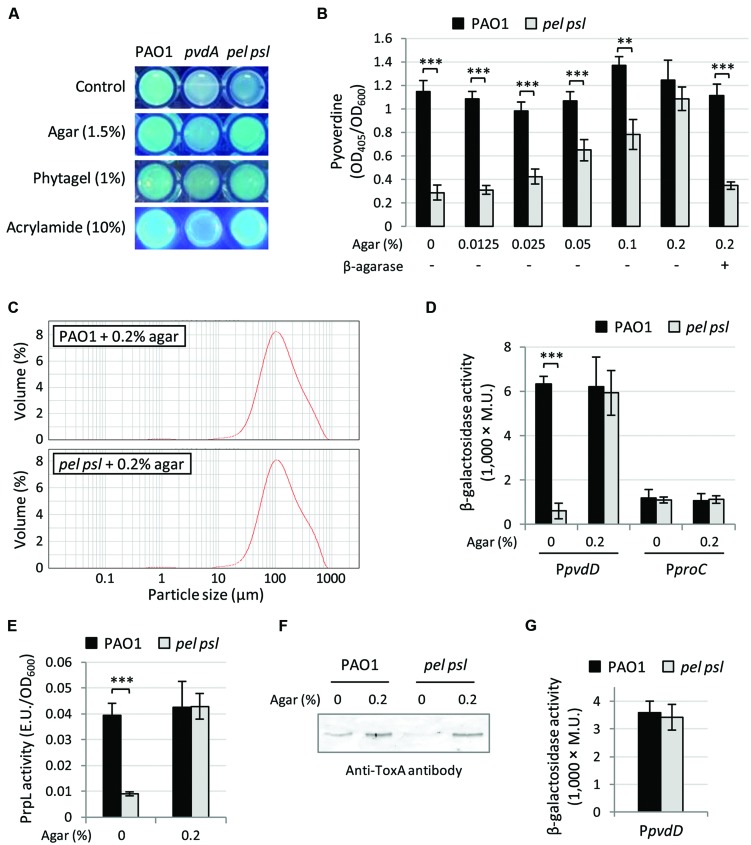
**Cell aggregation is involved in Pel and Psl-mediated control of pyoverdine-dependent virulence factors. (A)** Fluorescent phenotype upon UV light exposure of *P. aeruginosa* PAO1, the *pel psl* mutant and the *pvdA* mutant (used as pyoverdine-deficient negative control) grown in liquid TSBD medium (Control) or on TSBD solidified with 1.5% agar, 1% phytagel, or 10% polyacrylamide. **(B)** Pyoverdine production by *P. aeruginosa* PAO1 and the *pel psl* mutant grown in TSBD supplemented with increasing concentrations of agar (0–0.2%) and/or β-agarase I (3.3 units/ml). **(C)** Representative LDA particle-size scans of *P. aeruginosa* PAO1 and *pel psl* cultures in TSBD supplemented with 0.2% agar after 14 h of growth at 37°C. **(D)** Activity of the P*pvdD::lacZ* and P*proC’-‘lacZ* reporter fusions, **(E)** PrpL enzymatic activity and **(F)** ToxA levels in culture supernatants from *P. aeruginosa* PAO1 and *pel psl* cultures in TSBD supplemented or not with 0.2% agar. Bacteria were cultured in TSBD at 37°C under static conditions for 14 h. **(G)** Activity of the P*pvdD::lacZ* transcriptional fusion in *P. aeruginosa* PAO1 and *pel psl* cells cultured on TSBD solidified with 10% polyacrylamide. Values in **(B,D,E,G)** are the mean (±SD) of at least three independent assays. Asterisks indicate statistically significant differences with respect to the wild type strain (PAO1) grown under the same culture conditions (^∗∗^*p* < 0.01, ^∗∗∗^*p* < 0.001). Images in **(A,F)** are representative of two independent experiments giving similar results.

We thus hypothesized that cell-to-cell contacts and/or cell aggregation, instead of exopolysaccharides by themselves, could represent the signal triggering pyoverdine-dependent gene expression. This hypothesis was first tested by growing WT and *pel psl* mutant cells as colonies on the surface of TSBD medium solidified with different gelling substances, such as polyacrylamide and the polysaccharides agar and phytagel, and qualitatively assessing pyoverdine production by comparing fluorescence emission under UV light (Visca et al., 2007). While the fluorescence of the *pel psl* mutant growing as single cells in liquid medium was much lower than that of the aggregated WT and comparable to that of the pyoverdine-deficient mutant PAO1 *pvdA* (Imperi et al., 2008), the Pel and Psl-deficient mutant showed fluorescence levels that were indistinguishable from those of the WT, and much higher than those of the *pvdA* mutant, during aggregated (colony) growth on solid surfaces (**Figure 4A**). Although this assay clearly showed that growth on solid surfaces, where cells can interact with each other irrespective of aggregative polysaccharides, enhanced pyoverdine production in the *pel psl* mutant, the qualitative nature of the assay did not allow a quantitative comparison of pyoverdine levels between strains. To overcome this limitation, strains were cultured in TSBD medium supplemented with different sub-gelling concentrations of agar (0.0125–0.2%, **Figure 4B**) or phytagel (0.08–0.125%, Supplementary Figure S3), thus allowing to quantitatively assess pyoverdine levels in culture supernatants. Pyoverdine levels in the supernatants of *pel psl* mutant cultures were increased by sub-gelling concentrations of agar in a concentration-dependent manner, while agar had negligible effects on pyoverdine production by the WT strain (**Figure 4B**). Substantially similar results were obtained with phytagel (Supplementary Figure S3). LDA and microscopic analyses confirmed that the presence of sub-gelling concentrations of agar in the growth medium forced the *pel psl* mutant to grow as large clusters of cells comparable to those observed for the WT (**Figure 4C**), consistent with the conclusion that cell aggregation stimulates pyoverdine production.

Notably, pyoverdine production by the *pel psl* mutant in the presence of agar was abrogated by agar degradation with β-agarase I, which however, had no effect on pyoverdine production by WT PAO1 (**Figure 4B**). This result indicates that the effect of agar on the *pel psl* mutant is not related to any specific constituent of the polysaccharide matrix or to a general increase in the osmolarity of the growth medium. In fact, differently from agar and phytagel, high concentrations of sucrose (up to 10%) did not stimulate pyoverdine production by the exopolysaccharides-null mutant (Supplementary Figure S3). These evidences are in line with the finding that gelling agents only promoted pyoverdine production in the *pel psl* mutant but not in the WT (**Figure 4** and Supplementary Figure S3), indicating that this effect is specific to Pel and Psl-deficient cells. Coherent with the pyoverdine production profile, addition of 0.2% agar stimulated *pvdD* promoter activity (**Figure 4D**), as well as PrpL and ToxA production by the *pel psl* mutant (**Figures 4E,F**), while it had no effect on the expression of the housekeeping gene *proC* (**Figure 4D**). Notably, *pvdD* promoter activity in the *pel psl* mutant was also restored to WT levels by growing cells on the surface of TSBD solidified with polyacrylamide (**Figure 4G**), indicating that bacterial aggregation and/or cell contacts can trigger pyoverdine gene expression in the absence of any endogenous or exogenously added exopolysaccharide.

It appears therefore that artificially induced cell aggregation, obtained by growing cells either as colonies on solid surface or in liquid cultures in the presence of aggregating agents, is able to restore pyoverdine-dependent phenotypes in the Pel and Psl-deficient mutant, supporting the idea that cell-to-cell contacts and/or growth as aggregates are by themselves cues that stimulate pyoverdine production and virulence in *P. aeruginosa*.

### Contacts with Abiotic Surface and the Mechanosensors PilY1 and Type IV Pili are not involved in Aggregation-Mediated Activation of Pyoverdine Production

**FIGURE 5:**
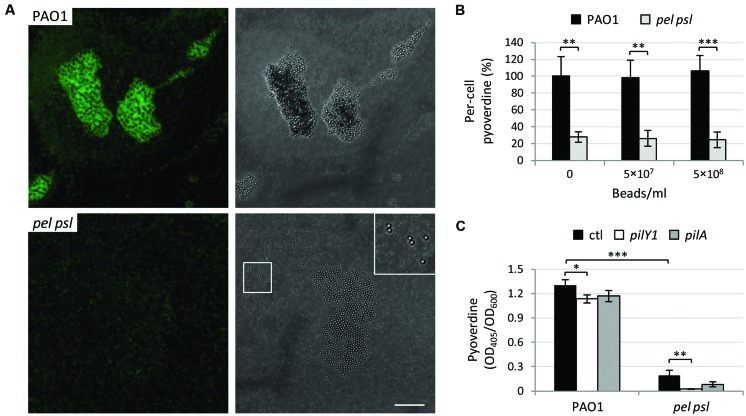
**Aggregation does not stimulate pyoverdine production thorough non-specific physical contacts or the mechanosensors PilY1 and type IV pili. (A)** Confocal microscopy images of *P. aeruginosa* PAO1 and *pel psl* cells harboring the GFP-expressing vector pMMG cultured in TSBD in the presence of 5 × 10^8^ polystyrene beads/ml. Images are representative of several micrographs showing similar results. Bar: 30 μm. The inset is a 2× magnification of the highlighted area. **(B)** Pyoverdine production by *P. aeruginosa* PAO1 and *pel psl* in the presence or in the absence of 3-μm size polystyrene beads (5 × 10^7^ or 5 × 10^8^ beads/ml), normalized to the number of colony forming units/ml and expressed as percentage with respect to the untreated wild type. Bacteria were grown in TSBD at 37°C for 14 h under vigorous shaking (220 rpm). **(C)** Pyoverdine production by *P. aeruginosa* PAO1, *pel psl* and their corresponding *pilY1* or *pilA* deletion mutants after 14 h of growth in TSBD at 37°C under static conditions. Values are the mean (±SD) of at least three independent assays. Asterisks indicate statistically significant differences with respect to the corresponding parental strain grown under the same culture conditions (^∗^*p* < 0.05, ^∗∗^*p* < 0.01, ^∗∗∗^*p* < 0.001).

In order to verify whether the stimulation of pyoverdine production by cell aggregation was due to specific cell-to-cell interactions or to an increase in surface contacts during growth as cellular aggregates, the *pel psl* mutant was cultured in the presence of 5 × 10^7^ or 5 × 10^8^ polystyrene beads per ml (3-μm size, Polysciences), under vigorous shaking in order to maintain beads in suspension. We calculated that the addition of 5 × 10^8^ beads/ml with a 3-μm diameter in the well of a 96-wells microtiter plate (containing 250 μl of medium) leads to >20-fold increase in the plastic surface available for cell contacts, relative to cultures without beads. While the WT strain formed large aggregates of bacterial cells around the beads, the *pel psl* mutant only attached to the beads without developing cell aggregates (**Figure 5A**), indicating that the presence of the beads actually increases the number of contacts between bacterial cells and the inert plastic material. However, polystyrene beads had no effect on pyoverdine production by the *pel psl* mutant or the WT used as control (**Figure 5B**), suggesting that cell aggregation, rather than non-specific surface contacts, represents the cue which triggers pyoverdine gene expression in *P. aeruginosa*.

To further verify this hypothesis, we investigated the possible involvement in aggregation-mediated induction of pyoverdine production of two *P. aeruginosa* mechanosensors, namely PilY1 and type IV pili, which have recently been implicated in surface contact-dependent activation of virulence gene expression (Siryaporn et al., 2014; Persat et al., 2015). Should PilY1 or type IV pili also be involved in pyoverdine activation in response to planktonic aggregation, the deletion of *pilY1* or *pilA* (encoding the major pilin subunit of type IV pili) should either reduce pyoverdine production in the WT or increase pyoverdine production in the *pel psl* mutant, in case these mechanosensors act as positive or negative aggregation-dependent regulators, respectively. However, deletion of *pilY1* or *pilA* caused only minor reduction (about 10%) of pyoverdine levels in the WT background, while it was not able to restore pyoverdine production in the *pel psl* mutant background (**Figure 5C**). This result indicates that formerly characterized mechanosensors, such as PilY1 and type IV pili, are not the effectors of aggregation-dependent activation of pyoverdine genes.

## Discussion

In the last decades, the old vision of bacteria as strictly unicellular organisms living in a planktonic single-cell status was swept away by the finding that bacterial cells in natural, industrial and many clinical settings predominantly exist as biofilms, i.e., structured microbial communities attached to a surface and encased in an extracellular matrix (Mann and Wozniak, 2012). Also during planktonic growth in liquid cultures bacteria can assemble into aggregates of densely packed cells, and it is believed that planktonic aggregation can play a role in resistance to stresses and antibiotics (Schleheck et al., 2009; Blom et al., 2010; Haaber et al., 2012), as well as in microbe–host cell interaction (Lepanto et al., 2011). Although hundreds of studies have investigated the physiology of biofilm-living bacterial cells, very little is known about the effects of cell aggregation during planktonic growth.

Here we provide evidence that growth as planktonic aggregates promotes production of three major virulence factors in the opportunistic pathogen *P. aeruginosa*, namely pyoverdine, extracellular protease PrpL and exotoxin A. Indeed, we observed that a Pel and Psl-null mutant unable to aggregate in liquid cultures is also defective in the expression of virulence factor genes, and that this effect can be rescued by artificially induced cell aggregation (**Figure 4** and Supplementary Figure S3).

Although in this study, we did not elucidate the mechanism by which cell aggregation is perceived by bacterial cells and translated into a transcriptional response which affects pyoverdine-related genes, two hypotheses can reasonably be made to explain the observed effect of planktonic aggregation on gene expression. First, a sensory machinery could transduce a contact signal deriving from the cell envelope into a cytoplasmic response. Our experiments lead to exclude the involvement of physical changes in the cell envelope due to contacts with abiotic surfaces, as well as any role of the two mechanosensors PilY and type IV pili (**Figure 5**), which have recently been found to induce *P. aeruginosa* virulence in response to surface contacts (Siryaporn et al., 2014; Persat et al., 2015). Also the *P. aeruginosa* Wsp system, a chemotaxis-like signal transduction complex which promotes c-di-GMP production by activating the diguanylate cyclase WspR, is stimulated by growth on surfaces (Güvener and Harwood, 2007). However, it has recently been shown that exopolysaccharides depletion in a c-di-GMP overproducing *P. aeruginosa* strain abrogated the ability of c-di-GMP to promote pyoverdine production (Chen et al., 2015). Accordingly, we found that the overexpression of a constitutively active WspR variant, which results in almost 100-fold increase in intracellular c-di-GMP levels, is unable to induce pyoverdine production in Pel and Psl-deficient cells (Supplementary Figure S4), reasonably excluding also the involvement of the Wsp system. However, other mechanosensitive factors or contact-dependent systems, which likely respond to specific cell-to-cell interactions, could exist in *P. aeruginosa*. For instance, the *P. aeruginosa* PAO1 genome is predicted to encode 26 methyl accepting chemotaxis proteins (Whitchurch et al., 2004), 13 cell–surface signaling systems (Llamas et al., 2014), up to 50 canonical two-component systems and more than 10 orphan sensor kinases (Gooderham and Hancock, 2009), many of them being implicated in virulence gene regulation.

The alternative hypothesis is that growth as aggregates determines changes in the cell surrounding environment which could influence virulence gene expression. Studies on the physiology of planktonic aggregates are still at an early stage, but it has recently been reported that localized oxygen depletion occurs in aggregates of *P. aeruginosa* cells grown in a gelatin-based microtrap (Wessel et al., 2014). Notably, oxygen is a well known inducer of pyoverdine gene expression (Ochsner et al., 1996; Llamas et al., 2014); thus oxygen limitation cannot explain the observed induction of pyoverdine production in bacterial aggregates. On the other hand, since exotoxin A expression is induced under microaerobic conditions through a PvdS-independent mechanism (Gaines et al., 2007), we cannot exclude that the increased exotoxin A expression levels in planktonic aggregates (**Figures 1** and **4**) could be related, at least in part, to oxygen depletion. It has also been reported that pyoverdine concentration is heterogeneous in *P. aeruginosa* microcolonies, with a maximum at the colony center (Julou et al., 2013). Although this heterogeneity could influence the efficiency of pyoverdine signaling and/or iron uptake in cell aggregates, we have demonstrated that the aggregation-mediated effect on pyoverdine production is independent of pyoverdine signaling and the iron sensor Fur (**Figure 2**), ruling out that increased virulence gene expression in aggregated cells may be due to altered pyoverdine signal transduction or dysregulated iron homeostasis. Thus, it is plausible that still uncharacterized chemical and/or physiological changes occurring in densely packed bacterial cells are responsible for the observed activation of pyoverdine-related virulence genes in *P. aeruginosa* planktonic aggregates.

In summary, our work provides the first evidence that formation of planktonic aggregates stimulates the production of pyoverdine-dependent virulence factors in *P. aeruginosa*. Further studies are necessary to clarify the mechanistic link between cell aggregation and activation of virulence gene expression. Such a kind of cell contact- or aggregation-dependent induction of virulence could represent a further strategy to modulate bacterial pathogenicity in response to population density, additional or complementary to chemical signaling via quorum sensing. Cellular aggregation also represents the first committed step of biofilm formation. Although some recent transcriptomics and proteomics studies highlighted an overall attenuation of virulence gene expression in mature *P. aeruginosa* biofilms (Li et al., 2014; Park et al., 2014), which include many slowly growing or quiescent cells, our finding could imply that the virulence potential of *P. aeruginosa* is increased during the first stages of biofilm formation, when siderophores, extracellular enzymes, and toxins would provide cells with essential nutrients for the energy-demanding process of biofilm development. This hypothesis is in line with the evidence that the gene expression profile of developing *P. aeruginosa* biofilms is more similar to that of exponential phase cultures rather than mature biofilms (Waite et al., 2006). Finally, since planktonic aggregation seems to be widespread among bacteria (Fazli et al., 2009; Schleheck et al., 2009; Blom et al., 2010; Haaber et al., 2012), it would be interesting to verify whether the correlation between cell aggregation and virulence is conserved in other bacterial pathogens.

## Author Contributions

Conceived and designed experiments: DV and FI. Performed the experiments: DV, MP, CB, and FI. Analyzed the data: DV, CB, VK, PV, and FI. Contributed reagents/materials/analysis tools: VK, PV, and FI. Wrote the paper: DV, PV, and FI. All authors read and approved the final manuscript.

## Conflict of Interest Statement

The authors declare that the research was conducted in the absence of any commercial or financial relationships that could be construed as a potential conflict of interest.
