# Supplementary material for: Cell aggregation promotes pyoverdine-dependent iron uptake and virulence in Pseudomonas aeruginosa
Source: Front Microbiol. 2015 Aug 28;6:902. doi: 10.3389/fmicb.2015.00902 (PMC4552172; doi:10.3389/fmicb.2015.00902)
Supplement: Supplementary file 1 [file Presentation_1.PDF]

Supplementary Material

Cell aggregation promotes pyoverdine-dependent iron uptake and virulence in *Pseudomonas aeruginosa*

Daniela Visaggio, Martina Pasqua, Carlo Bonchi, Volkhard Kaever, Paolo Visca, and Francesco Imperi*

*** Correspondence:** Francesco Imperi, francesco.imperi@uniroma1.it

1. Supplementary Figures and Tables

1.1. Supplementary Figures

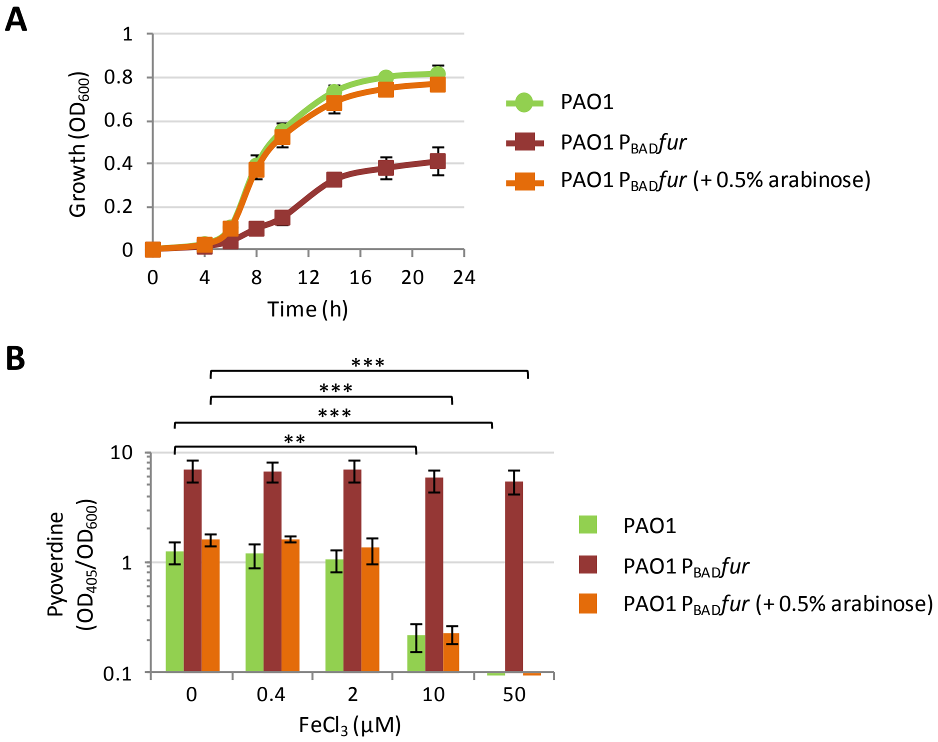

Supplementary Figure 1. Validation of the *fur* conditional mutant. (A) Growth of *P. aeruginosa* PAO1 and the *fur* conditional mutant (PAO1 P_{BAD}*fur*) in TSBD, supplemented or not with 0.5% arabinose, at 37°C in microtiter plates under static conditions, measured in Victor^{2}V plate reader (Perkin-Elmer). **(B)** Pyoverdine production in the *P. aeruginosa* PAO1 and the *fur* conditional mutant (PAO1 P_{BAD}*fur*) grown in TSBD, supplemented with increasing concentrations of $FeCl_3$ (0-50 µM) and/or 0.5% arabinose, at 37°C in microtiter plates under static conditions for 14 h. Values are the mean (± SD) of at least two independent assays. Asterisks indicate statistically significant differences with respect to the corresponding control grown in the absence of exogenously-added $FeCl_3$ (** p<0.01, *** p<0.001).

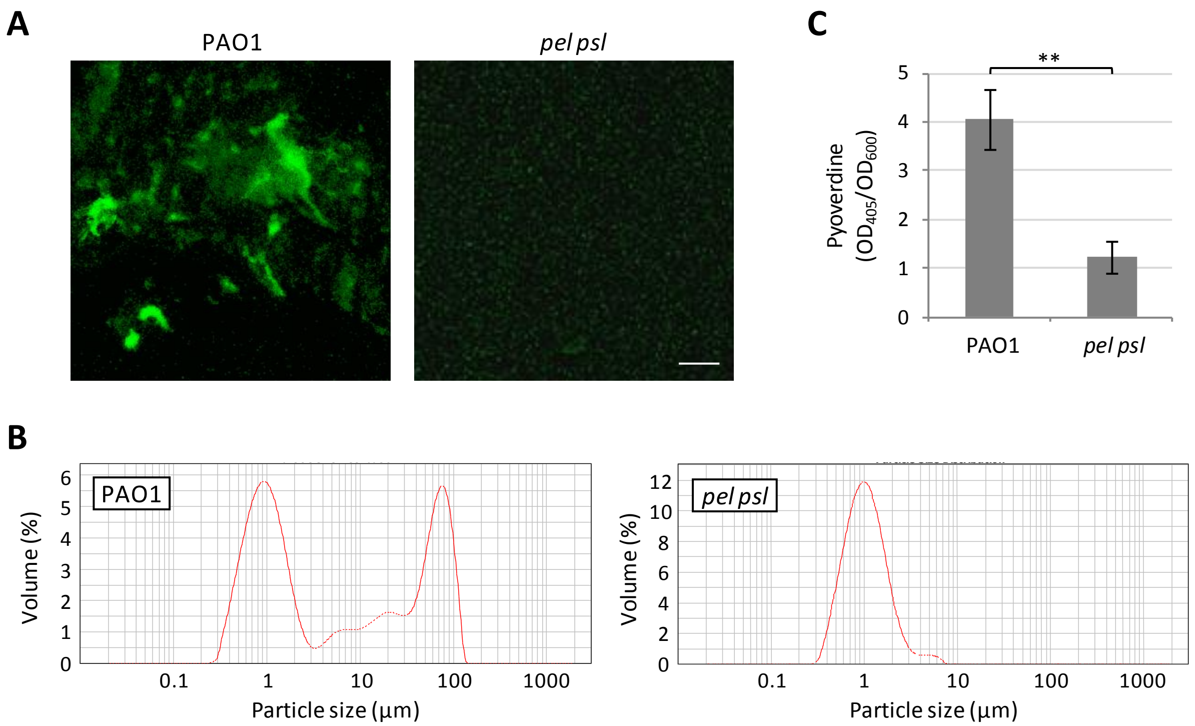

Supplementary Figure 2. Planktonic aggregation and pyoverdine production in M9 minimal medium. (A) Confocal microscopy images and **(B)** LDA particle-size scans of *P. aeruginosa* PAO1 and the *pel psl* mutant (harboring the GFP-expressing vector pMMG in the case of confocal microscopy analyses) grown for 18 h in M9 supplemented with 20 mM succinate at 37°C in microtiter plates under static conditions. Bar: 50 µm. **(C)** Pyoverdine production by *P. aeruginosa* PAO1 and the *pel psl* mutant cultured as described in panels A-B. Images are representative of at least two experiments giving similar results, while values in panel C are the mean (± SD) of three independent assays. Asterisks indicate a statistically significant difference ($p<0.01$).

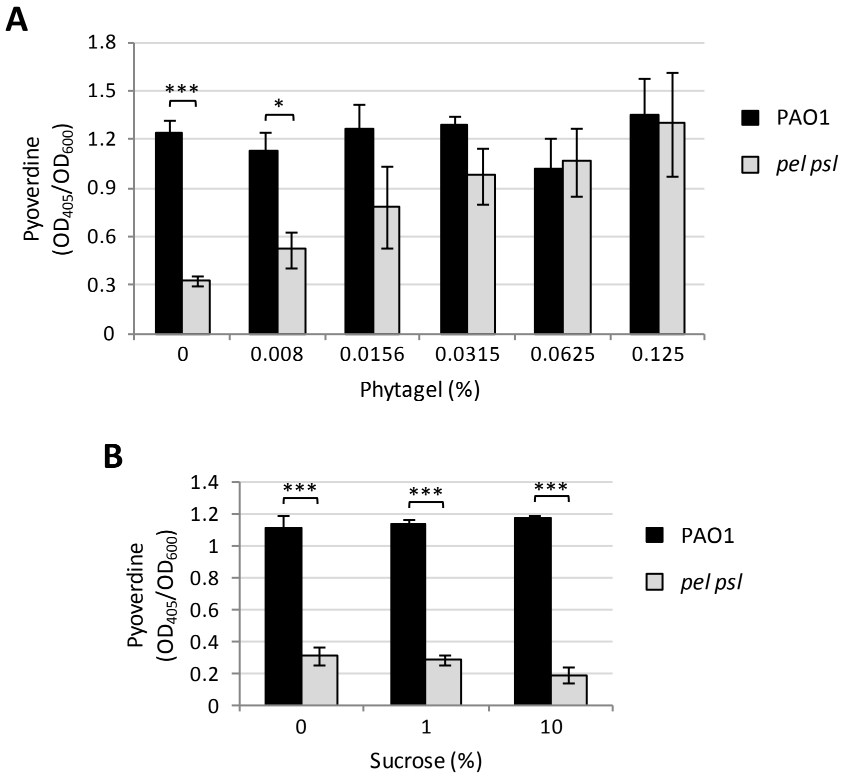

Supplementary Figure 3. Effect of phytagel or sucrose on pyoverdine production. Pyoverdine production by *P. aeruginosa* PAO1 and the *pel psl* mutant grown in TSBD at 37°C in microtiter plates under static conditions for 14 h, in the presence of increasing concentrations of **(A)** phytagel (0-0.125%) or **(B)** sucrose (0-10%). Values are the mean (± SD) of at least three independent assays. Asterisks indicate statistically significant differences with respect to the wild type grown under the same culture conditions (* $p<0.05$, *** $p<0.001$).

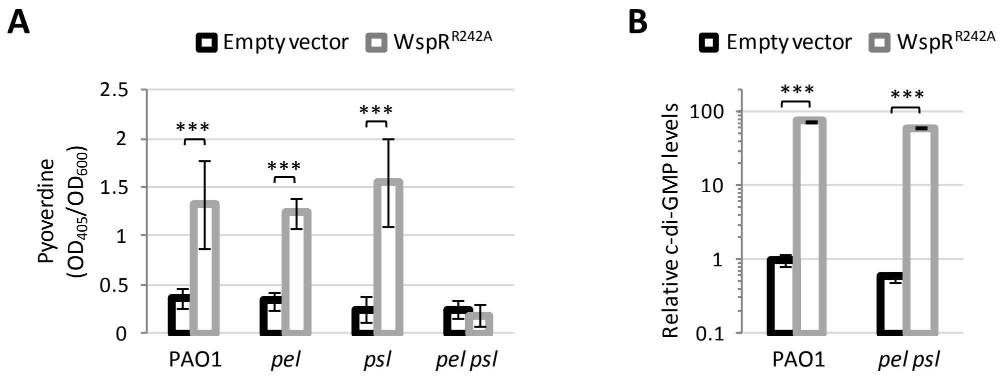

Supplementary Figure 4. (A) Pyoverdine production by *P. aeruginosa* PAO1, *pel*, *psl* and *pel psl* mutants harboring the empty vector pBBR1MCS-4 or its derivative constitutively expressing the diguanylate cyclase WspR^{R242A} (Moscoso *et al*., 2011; Table S1) grown in TSBD medium supplemented with 500 µg/ml carbenicillin for 14 h at 37°C at 200 rpm. **(B)** Intracellular levels of c-di-GMP (relative to PAO1 pBBR1MCS-4) in *P. aeruginosa* PAO1 and the *pel psl* mutant harboring pBBR1MCS-4 or its derivative constitutively expressing the diguanylate cyclase WspR^{R242A} cultured as describe in panel A. Values are the mean (± SD) of three independent assays. Asterisks indicate statistically significant differences with respect to the corresponding control carrying the empty vector (p<0.001).

1.2. Supplementary Tables

Supplementary Table 1. Bacterial strains and plasmids used in this study.

Strain or plasmid	Genotype and/or relevant characteristics	Reference or source
P. aeruginosa		
PAO1 (ATCC15692)	Prototroph	American type culture collection
PAO1 *rsmA*	PAO1 deleted of the *rsmA* coding sequence	Frangipani *et al.*, 2014
PAO1 *rsmY rsmZ*	PAO1 deleted of the *rsmY* and *rsmZ* genes	Frangipani *et al.*, 2014
PAO1 *pel*	PAO1 deleted of the *pelABCD* genes, including the promoter region	This work
PAO1 *psl*	PAO1 deleted of the *pslABCD* genes, including the promoter region	This work
PAO1 *pel psl*	PAO1 deleted of the *pelABCD* and *pslABCD* genes	This work
PAO1 *rsmA pel*	PAO1 deleted of the *rsmA* and *pelABCD* genes	This work
PAO1 *rsmA psl*	PAO1 deleted of the *rsmA* and *pslABCD* genes	This work
PAO1 *rsmA pel psl*	PAO1 deleted of the *rsmA*, *pelABCD* and *pslABCD* genes	This work
PAO1 *rsmY rsmZ pel*	PAO1 deleted of the *rsmY*, *rsmZ* and *pelABCD* genes	This work
PAO1 *rsmY rsmZ psl*	PAO1 deleted of the *rsmY*, *rsmZ* and *pslABCD* genes	This work
PAO1 *rsmY rsmZ pel psl*	PAO1 deleted of the *rsmY*, *rsmZ*, *pelABCD* and *pslABCD* genes	This work
PAO1 *fpvR*	PAO1 deleted of the *fpvR* coding sequence	This work
PAO1 *pel psl fpvR*	PAO1 deleted of the *pelABCD*, *pslABCD* and *fpvR* genes	This work
PAO1 *pilY1*	PAO1 deleted of the *pilY1* coding sequence	This work
PAO1 *pel psl pilY1*	PAO1 deleted of the *pelABCD*, *pslABCD* and *pilY1* genes	This work
PAO1 *pilA*	PAO1 deleted of the *pilA* coding sequence	This work
PAO1 *pel psl pilA*	PAO1 deleted of the *pelABCD*, *pslABCD* and *pilA* genes	This work
PAO1 *fur araC*P_{BAD}*fur*	PAO1 deleted of the *fur* coding sequence and carrying an arabinose-dependent copy of *fur*	This work
PAO1 *pel psl fur araC*P_{BAD}*fur*	PAO1 *fur araC*-P_{BAD}*fur* deleted of *pslABCD* and *pelABCD* genes	This work
PAO1 *pvdA*	PAO1 deleted of the *pvdA* coding sequence	Imperi *et al.*, 2008
PAO1 *toxA*	PA01 mutant with a transposon insertion in *toxA* (Mutant ID 40695), Tc^R	Jacobs *et al.*, 2003
E. coli		
S17.1λ*pir*	*thi pro hsdR hsdM*$^+$ *recA RP4-2-Tc::Mu-Km::Tn7 λpir,* Gm^R	Simon *et al.*, 1983

DH5αF’	*recA1 endA1 hsdR17 supE44 thi-1 gyrA96 relA1* Δ(*lacZYA-argF*)U169[ϕ*80 dlacZ*Δ*M15*], Nal^R	Liss, 1987
**Plasmid**		
pBluescript-II KS+	Cloning vector; ColE1 replicon; Ap^R	Stratagene
pDM4	Suicide vector; *sacBR, oriR6K*; Cm^R	Milton *et al.*, 1996
pDM4Δ*pelABCD*	pDM4 derivative for *pelABCD* deletion	Leoni L., unpublished
pDM4Δ*pslABCD*	pDM4 derivative for *pslABCD* deletion	Leoni L., unpublished
pDM4Δ*fpvR*	pDM4 derivative for *fpvR* in-frame deletion	Visca P., unpublished
pDM4Δ*fur*	pDM4 derivative for *fur* in-frame deletion	This work
pDM4Δ*pilY1*	pDM4 derivative for *pilY1* in-frame deletion	This work
pDM4Δ*pilA*	pDM4 derivative for *pilA* in-frame deletion	This work
mini-CTX1	Self-proficient integration vector with *tet*, Ω-*FRT-attP*-MCS, *ori, int,* and *oriT*; Tc^R	Hoang *et al.*, 2000
mini-CTX1-*araC*P_{BAD}*tolB*	mini-CTX1 derivative carrying *araC*P_{BAD}*tolB*	Lo Sciuto *et al.*, 2014
mini-CTX1-*araC*P_{BAD}*fur*	mini-CTX1-*araC*P_{BAD}*tolB* derivative in which *tolB* has been replaced with *fur* by HindIII/EcoRI digestion	This work
pBBR1MCS-4	Broad-host range cloning vector; Ap^R	Kovach *et al.*, 1995
pBBR1MCS-4-*wspR*^{R242A}	R252A-*wspR* cloned into pBBR1MCS-4	Moscoso *et al.*, 2011
pME3641	Plasmid carrying a translational P*proC*::*lacZ* fusion, Cb^R	Savioz *et al.*, 1993
pMP190::P*pvdD*	Plasmid carrying a transcriptional P*pvdD*::*lacZ* fusion, Cm^R	Cunliffe *et al.*, 1995
pMMG	pME6032 derivative constitutively expressing the green fluorescent protein (GFP), Tc^R	Popat *et al.*, 2012

Supplementary Table 2. Primers used in this study.[1]

Primer name	Sequence (5’-3’)[2]	Restriction site	Application
*fur*_FW	CCCAAGCTTATGGTTGAAAATAGCGAACTTC	HindIII	Generation of the mini-CTX1-*araC*P_{BAD}*fur* construct
*fur*_RV	CGGAATTCATGGAACCGTTGCGCGAC	EcoRI	Generation of the mini-CTX1-*araC*P_{BAD}*fur* construct
fur mut_UP_FW	CCGCTCGAGTCGGAACCGGTACCG	XhoI	Generation of the pDM4Δ*fur* construct
fur mut_UP_RV	CGGGATCCATGTCTGCTTTCTCAGCG	BamHI	Generation of the pDM4Δ*fur* construct
fur mut_DOWN_FW	CGGGATCCGCGCGGCTTCGAGCTGG	BamHI	Generation of the pDM4Δ*fur* construct
fur mut_DOWN_RV	GCTCTAGAGTTCCTGGTCAGCGCC	XbaI	Generation of the pDM4Δ*fur* construct
pilY1 mut_UP_FW	CCGCTCGAGCATGCGCGAAGTG	XhoI	Generation of the pDM4Δ*pilY1* construct
pilY1 mut_UP_RV	CGGGATCCGGCGTGGGTGGTCTGGGC	BamHI	Generation of the pDM4Δ*pilY1* construct
pilY1 mut_DOWN_FW	CGGGATCCTCGGGCGAATGCC	BamHI	Generation of the pDM4Δ*pilY1* construct
pilY1 mut_DOWN_RV	GCTCTAGACTGGTAACTGGGAATGGCG	XbaI	Generation of the pDM4Δ*pilY1* construct
pilA mut_UP_FW	CCGCTCGAGCTTGTTGCCGCGCCG	XhoI	Generation of the pDM4Δ*pilA* construct
pilA mut_UP_RV	CGGGATCCTTTCATGAATCTCTCCGTTG	BamHI	Generation of the pDM4Δ*pilA* construct
pilA mut_DOWN_FW	ACCCAGGATCCGATGTTCAC	BamHI	Generation of the pDM4Δ*pilA* construct
pilA mut_DOWN_RV	GCTCTAGAACCTCGATGAACTGCGC	XbaI	Generation of the pDM4Δ*pilA* construct
pel mut_UP_FW	ATATCTAGAACGCCGTTACGGCACCCT	XbaI	PCR check of *pel* deletion mutants
pel mut_DOWN_RV	ATACTCGAGGGGCGAAGAGAATCCTCAG	XhoI	PCR check of *pel* deletion mutants
psl mut_UP_FW	ATATCTAGACCGAAATGGCACGAGGCG	XbaI	PCR check of *psl* deletion mutants
psl mut_DOWN_RV	ATACTCGAGTCAGCGATCATTGTTGACGG	XhoI	PCR check of *psl* deletion mutants
fpvR mut_UP_FW	GCTCTAGAAGGAACTGCGGCAGATG	XbaI	PCR check of *fpvR* deletion mutants
fpvR mut_UP_RV	CCGCTCGAGGGTGTACTGGGCAC	XhoI	PCR check of *fpvR* deletion mutants
*rpsL*_RT_FW	AAGCGCATGGTCGACAAG		Real-time PCR
*rpsL*_RT_RV	TACGCTGTGCTCTTGCAGG		Real-time PCR
*pvdD*_RT_FW	GAAAGGAAGGCATTGGCTG		Real-time PCR
*pvdD*_RT_RV	GTAGACGCAAGACACTCGGG		Real-time PCR
*toxA*_RT_FW	CGACCTCTGGAACGAATGC		Real-time PCR
*toxA*_RT_RV	TTGTCGATGGCCAGCTTG		Real-time PCR
*prpL*_RT_FW	GCCGGCAAGGAAATCTTC		Real-time PCR
*prpL*_RT_RV	CAGGGAGTCGGCGAAATAC		Real-time PCR

2. **Additional references** (not included in the main text)

Cunliffe, H.E., Merriman, T.R., and Lamont, I.L. (1995). Cloning and characterization of *pvdS*, a gene required for pyoverdine synthesis in *Pseudomonas aeruginosa*: PvdS is probably an alternative sigma factor. *J. Bacteriol.* 177, 2744–2750.

Hoang, T.T., Kutchma, A.J., Becher, A., and Schweizer, H.P. (2000). Integration-proficient plasmids for *Pseudomonas aeruginosa*: site-specific integration and use for engineering of reporter and expression strains. *Plasmid* 43, 59-72.

Jacobs, M.A., Alwood,A., Thaipisuttikul, I., Spencer, D., Haugen, E., Ernst, S., *et al.* (2003). Comprehensive transposon mutant library of *Pseudomonas aeruginosa*. *Proc. Natl. Acad. Sci. U.S.A.* 100, 14339-14344.

Kovach, M.E., Elzer, P.H., Hill, D.S., Robertson, G.T., Farris, M.A., Roop, R.M.I., *et al.* (1995). Four new derivatives of the broad-host-range cloning vector pBBR1MCS, carrying different antibiotic-resistance cassettes. *Gene* 166, 175–176.

Liss, L. (1987). New M13 host: DH5 F′ competent cells. *Focus* 9, 13.

Milton, D.L., O'Toole, R., Horstedt, P., and Wolf-Watz, H. (1996). Flagellin A is essential for the virulence of *Vibrio anguillarum*. *J. Bacteriol.* 178, 1310-1319.

Popat, R., Crusz, S.A., Messina, M., Williams, P., West, S.A., and Diggle, S.P. (2012). Quorum-sensing and cheating in bacterial biofilms. *Proc. Biol. Sci.* 279, 4765-4771.

Savioz, A., Zimmermann, A., and Haas, D. (1993). *Pseudomonas aeruginosa* promoters which contain a conserved GG-N10-GC motif but appear to be RpoN-independent. *Mol. Gen. Genet.* 238, 74-80.

Simon, R., Priefer, U., and Pühler, A. (1983). A broad host range mobilization system for *in vivo* genetic engineering: transposon mutagenesis in Gram negative bacteria. *Bio/Technology* 1, 784-790.
